# Prevalence, causes, and complications of cesarean delivery in Iran: A systematic review and meta-analysis

**Published:** 2018-04

**Authors:** Mohammad Rafiei, Marzieh Saei Ghare, Malihe Akbari, Faezeh Kiani, Fatemeh Sayehmiri, Koroush Sayehmiri, Reza Vafaee

**Affiliations:** 1 *Department of Biostatistics and Epidemiology, School of Medicine, Arak University of Medical Sciences, Arak, Iran. *; 2 *Student Research Committee, Midwifery and Reproductive Health Research Center, school of Nursing and Midwifery, Shahid Beheshti University of Medical Sciences, Tehran, Iran. *; 3 *Department of Nursing and Midwifery, Ilam University of Medical Sciences, Ilam, Iran. *; 4 *Student Research Committee, Ilam University of Medical Sciences, Ilam, Iran. *; 5 *Proteomics Research Center, Shahid Beheshti University of Medical Sciences, Tehran, Iran. *; 6 *Department of Biostatistics, Faculty of Medicine, Ilam University of Medical Sciences, Ilam, Iran. *

**Keywords:** Cesarean, Iran, Prevalence, Meta-analysis

## Abstract

**Background:**

Uncontrolled increase of C-section is one of the major problems in Iranian health system, such that C-section is the most common surgical procedure in the entire country’s hospitals in Obstetrics and Gynecology sections. A variety of complications also come along with cesarean.

**Objective:**

The aim of this study was to evaluate the prevalence, causes, and complications of cesarean in Iran.

**Materials and Methods:**

forty-one articles were considered with respect to certain criteria and were included in a systematic review to perform a meta-analysis study. The systematic review’s search was conducted on SID, Iranmedx, Magiran, Medlib, PubMed, and Science Direct databases published between1999-2016. The weight of each included study was calculated according to its sample size and the reported prevalence of binomial distribution. A random-effects model using R and STATA (Version 11.2) software was utilized for analyzing data

**Results:**

The total number of the sample was 197514 pregnant women with a mean age of 26.72 yr. The prevalence of cesarean in Iran was estimated at 48%. The main reasons for the prevalence of cesarean in this study were mothers’ higher education, previous cesarean, and doctor recommendation. The most frequent complication in women undergoing cesarean was the muscular pain, and the most common fetal complications in newborns by caesarean delivery was transient tachypnea.

**Conclusion:**

The prevalence of C-section in Iran is much higher than what WHO recommends. It is essential, to decrease such a phenomenon, making the mothers aware of the risks of cesarean delivery, and establishing counselling sessions as well to eliminate the mothers’ fear of vaginal delivery.

## Introduction

Once upon a time in the last century, the modern cesarean delivery was begun to reduce the maternal and newborns complications, morbidity and mortality (1). Unfortunately nowadays, however, undergoing cesarean is not used only when necessary and only to save the mother and the baby; rather, it is gradually being assumed as something luxurious by some communities (2). In almost all of the scientific resources, the expected rate of cesarean delivery is considered as low as 13%, and according to the World Health Organization documents, it is recommended to be as low as 15% (3). Those documents also report the average rate of cesarean delivery in recent years has increased by 10-15% in the entire world’s countries. Some studies show that the probability that a woman undergoes a cesarean is 3 times more than that of 20 yr ago (4). The increasing caesarean section (C-section) has also been different in different countries (5), such that for developing countries it is much more than for developed ones. For example, caesarean rate in Brazil, Chile and China has increased up to 40-42% (6, 7). while, the rate of cesarean in Iran been reported from 26- 66.5% by various studies and as 87% by some private centers (8, 9). Cesarean delivery is carried out due to such various reasons as pregnancy at higher ages, lower number of a woman previous pregnancies, obesity, fetal distress, etc (10, 11). The most common reason cited for cesarean delivery in Iran, unlike the abovementioned reasons, is the previous cesarean (12). So, the main reason for the high rates of cesarean in Iran is an increased incidence of elective cesareans which are operated with no etiology just upon the patients’ request. According to some investigations, the main reason of elective cesareans in Iran is the fear of labor’s pain (8, 13). However, there are also other factors which affect the excessively increasing rate of cesarean in Iran such as people’s education, occupation, age, and place of residence (14, 15). 

Cesareans without indications, as compared to Normal Vaginal Delivery (NVD), would bring about many complications for both mother and the baby (16, 17). In addition, the results of studies in the UK have shown that the risk of maternal death caused by cesarean delivery is 3 times more than that of NVD (18). Many people think there is more probability of newborns health in case of cesarean, while studies have shown that the risk of death in newborns by cesarean is 4 times as much as newborns born by NVD (19). The most serious complications for the babies born by cesarean are fetal respiratory problems such as Transient Tachypnea (TTN) and Respiratory Distress Syndrome (RDS), surgical blade cuts, and increased rates of newborns admission in the neonatal intensive care unit (20-22). Also, experts believe that 1 min Apgar score of the newborns by cesarean is less that of the newborns by NVD (23). 

Given that today, excessively increasing rate of cesarean section is one of the major problems in Iranian health system, numerous studies have been conducted in this field in Iran in the last two decades. Despite repeated statements made by the national media and medical records, few attempts have been made to identify the extent of the problem and to identify possible causes and complications. However, recently a meta-analytical study has been carried out on the prevalence and causes of cesarean section in Iran (24), but this article is based on studies conducted until 2011, also in the present study, in order to reduce the time effect, the articles published in the period 1999-2016 was investigated. Since the changes in the prevalence of C-section and its complications are very important, it was necessary to update the previous meta-analysis study. 

The aim of this study was meta-analysis evaluation of the prevalence, causes, and complications of cesarean in Iran.

## Materials and methods

This paper evaluates the prevalence, causes, and complications of cesarean in Iran using documentations review via a meta-analysis of the available resources within a period since oct 1999 to December 2016, this period was chosen consciously to reduce the time effect. Because, the time effect can be influenced by the changes in medical facilities and developments, community awareness and mothers attitude, on the prevalence and causes of cesarean section as well as maternal and neonatal complications due to C-section. So, choosing that period would help to make the results more realistic.

This studies contains several sections including accurate determination of the variables, data collection, data analysis, and interpreting the results. A variety of national and international scientific journals and scientific databases, such as PubMed, Iranmedx, SID, Medlib, Science Direct, Magiran were searched to find the results of conducted studies as well as papers presented in the relevant Iranian and international seminars and conferences. Search for the articles was carried out mainly through systematic searching for the valid keywords like “cesarean, delivery, prevalence, neonatal complications, maternal complications, Iran” as well as all their possible combinations both in Persian and English.

**Article selection**

At first, all papers contained in the aforementioned databases were independently evaluated by the researchers to identify and select the relevant titles and abstracts. Then, the selected articles were independently entered into the research process. The main criterion for any study to be included in the research was referring to either of “cesarean prevalence”, “neonatal complications”, “maternal complications”, and “Iran” in its title or abstract. Studies which were not included in the preliminary studies or those on the areas unrelated to the subject of the present study (i.e. the research with the subject of cesarean, but without examining the causes and complications of cesarean delivery, and qualitative studies) were excluded. 

Having determined the relevant studies in terms of their titles, the abstracts of the selected articles using were evaluated the STROBE checklist. The selected articles were fully investigated and all their information was entered in a form designed to extract the data. Then, the data were entered into Excel software. Subsequently, the data were moved from Excel into R and STATA software (version 11.2). 

A Begged funnel plot and also the “trim and fill” method were used for correcting publication bias. The “Trim and fill” method, which makes strong symmetric assumptions, beside a sensitivity analysis, which is a cautious approach, make the conclusion of meta analyze under several plausible possibilities. regarding the extent of publication bias, the conclusion would be different between standard approach and the other approaches (25). 

**Statistical analysis**

Given that the main criteria in this study were the prevalence of cesarean, its variance (with 95% confidence interval (CI)) was calculated using the binomial distribution. The weight of each study was considered as the reciprocal of its variance and then was used to participate in combining the reported prevalence of C-section from the selected studies to calculate a weighted average of the general C-section rate. Q test and I^2^ index with α significance level of less than 10% were used to investigate heterogeneity. Heterogeneous studies, if they existed, were analyzed using a meta-analysis model with random effects. R and STATA software (version 11.2) was used to analyze the data.

## Results

**Figure 1:**
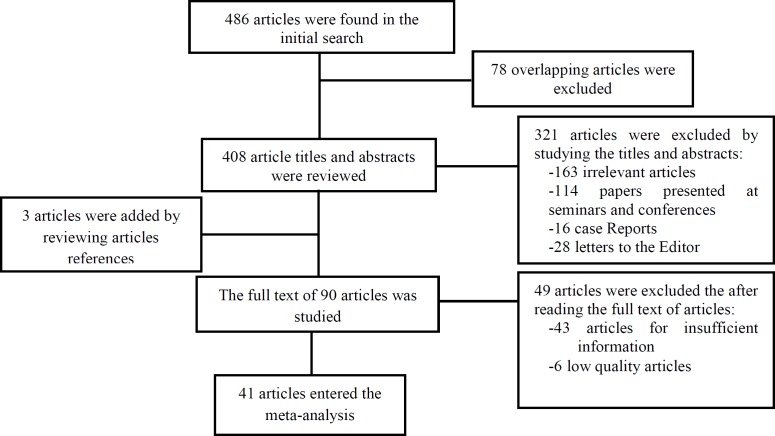
Flow diagram of prevalence, causes, and complications of cesarean delivery in Iran

Having eliminated the repeated and irrelevant studies, 41 articles remained with the objectives of the research which were selected and examined (Appendix 1). The study selection process is shown in Figure 1.

All the studies examined were cross-sectional researches. In these studies, the sampling method was census; hospital profiles and questionnaires were used for data collection which was completed through interviews and observations. The total number of the sample was 197514 pregnant women with a mean age of 26.82±5.13 yr old, out of whom 94807 women (48%) had undergone cesarean delivery and 102707 (52%) had had a labor through NVD. The most frequent studies were for Tehran province (25.6%). The lowest incidence of cesarean was related to a study by Soleimanizadeh and colleagues in Bam (13%) (26), and the highest incidence of cesarean was reported in a study by Azizi in Tehran (86%) (27). From a total of 44 articles entered into the study, 41 were included in the meta-analysis. 

**Figure 2:**
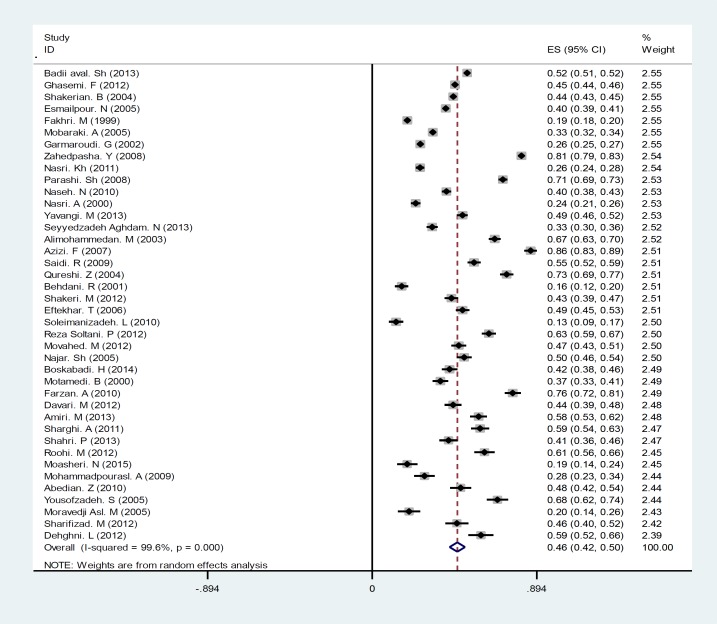
The prevalence of cesarean delivery in Iran based on random effects model. The midpoint of each segment is the estimate of prevalence and segment lengths show the 95% CI for each study. The diamond mark shows the prevalence in the country for all studies

**Figure 3:**
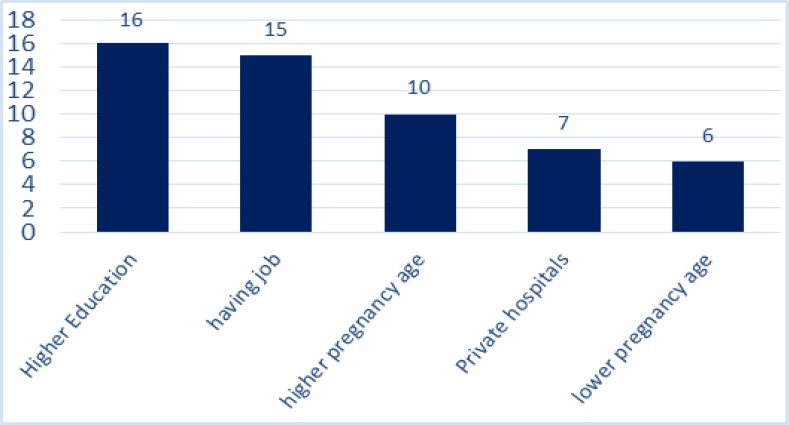
The frequency of socio-demographic factors affecting the incidence of caesarean (Y axis: percent

**Figure 4:**
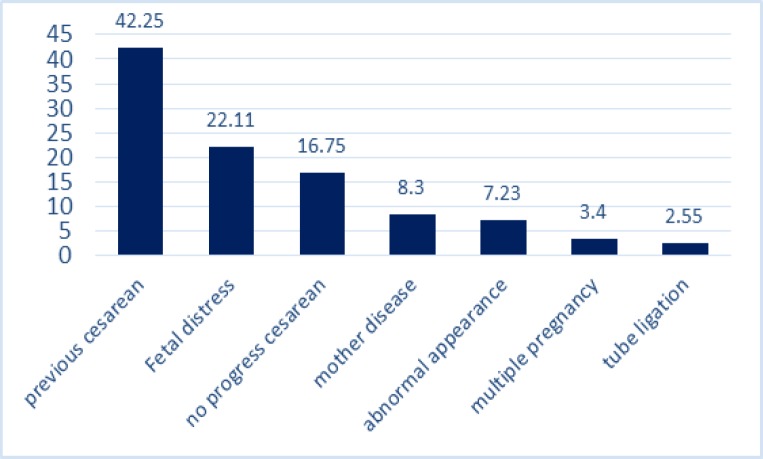
The frequency of obstetric-medical factors affecting the incidence of cesarean delivery

A Cochran’s Q test showed the heterogeneity of the studies’ findings (I2=100 %) so that a random effect model was used in all the subsequent stages. The prevalence of cesarean with CI of 95% for all studies in all regions of Iran is shown in Figure 2. Based on random effect model, cesarean rate in Iran was calculated as 48% (95%CI=36-59%). Individual and social variables affecting the incidence of caesarean section, in this study, using repetition frequency in studies is shown in Table I. Through content analyzing the reasons of occurrence of cesarean section were divided into two sets: 1) medical reasons 2) Non-medical reasons. The impacts of any of the risk factors of cesarean delivery, as the mean of the percentages, are shown in Table I. As seen in Figure 3, mothers’ higher education and mothers’ employment with 16 and 15 repetitions, were taken into consideration as an important socio-demographic factor for the prevalence of cesarean in the reviewed articles. High pregnancy age (>35 yr) was also another factor affecting the incidence of cesarean mentioned in the articles. The first set of factors affecting cesarean is medical reasons including a condition that the health of the mother or fetus is endangered in case of surgical intervention. The mean of any of these reasons is shown in Figure 2. As it can be seen in Figure 4, among the reasons studied in this research, previous cesarean (42.25%), reduced fetal movements (2.1%) and fetal distress (22.11%) are the main medical reasons for cesarean delivery. Also among the reviewed medical reasons, tubal ligation (2.55%) and multiple pregnancies (3.2%) are the least affecting factors. The second set of factors affecting the prevalence of cesarean examined in this study was including non-medical reasons. Such reasons are most commonly associated with the attitude and knowledge of families and doctors and mentality and attitude of the individuals. These factors are shown in Figure 3.

**Figure 5:**
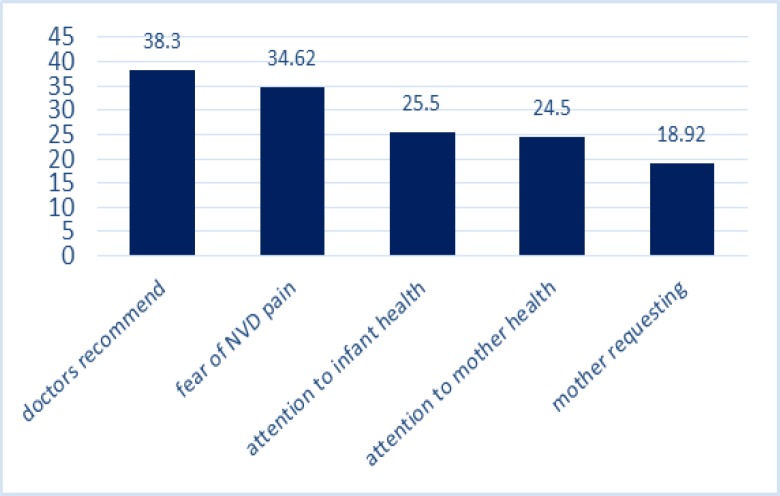
The frequency of non-obstetric-medical factors affecting the incidence of cesarean delivery

**Figure 6:**
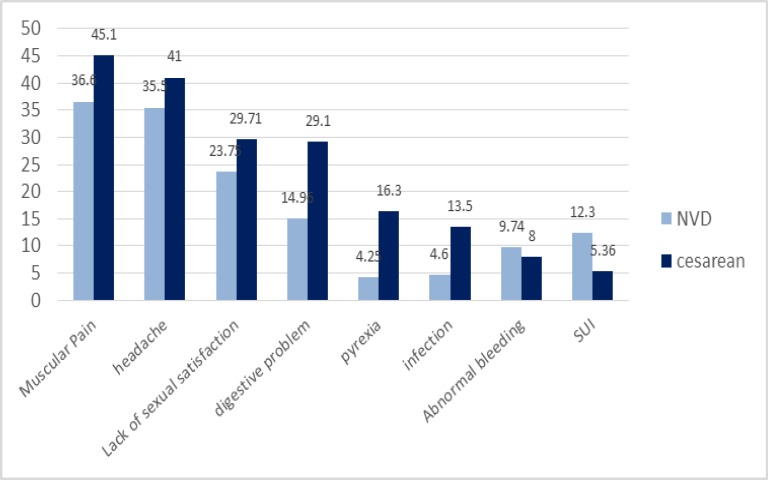
Comparison of maternal complications in cesarean and vaginal delivery

**Figure 7:**
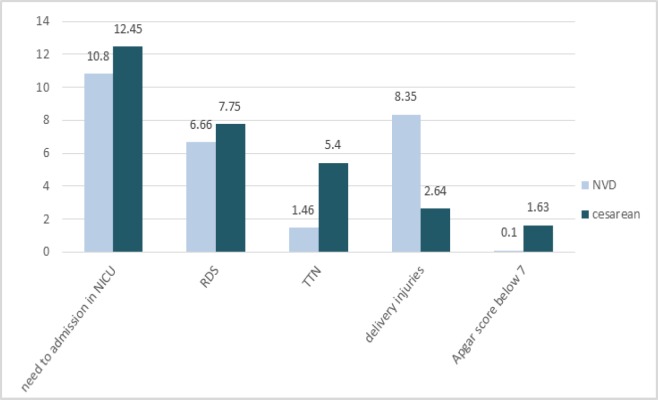
Comparison of neonatal complications in infants born by cesarean and vaginal delivery

As shown in Figure 5, doctor recommendation (38.8%) and mothers’ fear of vaginal delivery (34.62%) are the most important non-medical factors affecting the cesarean prevalence rate. Also among non-medical reasons examined it closely with an average of 13.3%, the lowest factor affecting cesarean section. Common complications resulting from the cesarean delivery were also divided into two categories of maternal and neonatal complications, in order to be examined separately. For a better comparison of cesarean complications with those of vaginal delivery in both mothers and the newborns, the average of the percentages mentioned for both cesarean and vaginal delivery complications included in this study are presented in figures 6 and 7. As shown in Figure 6, the average frequency of such maternal complications as muscular pain, headache, lack of sexual satisfaction after delivery, digestive problems, fever and infection in women who had undergone cesarean section is higher than that of the women who had had normal virginal delivery. Abnormal bleeding and stress urinary incontinence is also higher in the cesarean group. In addition, muscular pain (45.1%) and headache (41%) are the most frequent complications of caesarean section for the mothers. Moreover, the average maternal admission was 1.65 days for the virginal delivery and 3.1 days for the cesarean group. As it can be seen in figure 7, the Neonatal Intensive Care Unit (NICU: 12.45%) and Respiratory Distress Syndrome (RDS: 7.75%) the most common cesarean newborn complications. The frequency of NICU, RDS, TTN, and below-7-Apgar score in cesarean newborns were also higher than those of normal vaginal newborns. In contrast, labor injuries to the newborns are more common during vaginal delivery than during cesarean delivery (Table I). Meta-regression model demonstrated an increasing trend in prevalence of cesarean in Iran during 2000 till 2015.

**Publication bias**

**Figure 8:**
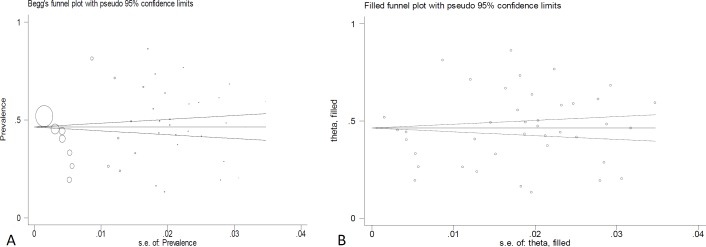
A) Funnel plot for checking publication bias. B) Trim and filled analysis for publication bias

Publication bias was checked using a Begg’s funnel plot. To check the large study bias at first, the studies were sorted from the most precise to the least precise (according to standard error), and then a cumulative random effect meta-analysis was run to realize if there was any trivial change in effect size (Figure 8.A). Trim and fill analysis was done as well to check the effects of missing study on the overall results. Duval and Tweedie’s Trim and Fill analysis showed that, the effect of missing studies keep results unchanged (Figure 8.B). An Egger’s test also showed a non-significant effect of publication bias (t=-0.64, p=0.527)

**Sensitivity analysis**

Sensitivity analysis was done to find the effect of influential studies on the overall results. There were no any study to change the overall results.

**Table I:** Frequency of individual and social variables, medical and non-medical factors affecting the prevalence of cesarean section

**Factor **	**Frequency (%)**
Individual and social variables	
Number of pregnancy	2
Low age of mother	3
Husband education	3
Marriage age	4
Location	4
Class status	5
Lack of knowledge	6
Private hospital	7
Mother old age	10
Maternal employment	12
Academic education	16
Medical factors	
Decrease Fetal movement	2.1
Twain	3.2
History of abortion	3.4
History of infertility	3.8
Preeclampsia	4.2
Tubal ligation	4.5
Abnormal presentation	7.23
Maternal disease	8.3
CPD	8.75
Failure of labor progression	16.75
Fetal distress	22.11
Repeated Cesarean	42.25
Non-medical factors	
Recommended acquaintances	13.3
Husband suggests	14.8
Mother suggests	19.1
Preserve the mother's health	24.5
Preserve the fetal's health	25.5
Fear of pain	34.62
Doctor suggest	38.3

CPD: Cephalopelvic disproportion

**Table II.** Prevalence of cesarean based on the time course and province

			**Prevalence of cesarean ** **% (95% CI)**	**Number of studies**
Time course				
	1999-2004		38 (27-49)	8
	2005-2009		53 (41-65)	11
	2010-2014		50 (34-65)	21
	2015-2017		19 (14-24)	1
Province				
		Chaharmahal and Bakhtiari	44 (43-45)	1
		Ardebil	59 (54-63)	1
		East Azerbaijan	28 (23-34)	1
		Gilan	52 (29-74)	2
		Hormozgan	24 (21-26)	1
		Isfahan	76 (72-81)	1
		Khorasan	47 (37-57)	5
		Kerman	45 (23-68)	5
		Khuzestan	46 (37-54)	2
		Kohgiluyeh and Boyer-Ahmad	33 (32-34)	1
		Markazi	29 (23-36)	2
		Mazandaran	49 (0.2-97)	3
		Samnan	58 (53-62)	1
		Fars	47 (43-51)	1
		Tehran	47 (33-62)	10
		West Azerbaijan	61 (56-66)	1
		Yazd	45 (44-46)	1
		Zanjan	43 (39-47)	1

**Appendix 1.** Summary of papers reviewed in this study

**Cesarean complications**	**Listed items Cesarean causes**	**Prevalence** ** (%)**	**Year**	**Sample Size**	**Source**
	Fetal distress, lack of labor progression, fear of pain, previous Cesarean , tubal ligation, History of infertility, miscarriage and stillbirth, maternal blood pressure	43	2012	697	(4)
TTN, RDS, labor injuries,NICU admission					
	Lack of labor progression, previous Cesarean , abnormal presentation, doctor recommendation, mother request, multiple pregnancy, private hospitals, higher education, working mothers, CPD ,decreased fetal movement, blood pressure	66.5	2003	824	(13)
	Lack of labor progression, previous Cesarean , abnormal presentation, fetal distress, maintaining the health of both the mother and fetus	23.6	2000	1080	(15)
TTN، RDS		81	2008	2023	(22)
	Mother suggest	43.9	2012	459	(28)
	Spouse's education, lack of awareness	41.9	2014	392	(31)
	Private hospitals, previous Cesarean , higher education, working mothers, gravidity	28.4	2009	250	(35)
	Private hospitals, previous Cesarean , mother request, Lack of knowledge	76.3	2010	360	(36)
Fever, muscular pain, infection, labor injuries	Fetal distress, lack of labor progression, maintaining the health of both the mother and fetus, previous Cesarean , previous Cesarean , higher education, working mothers, high pregnancy age	40.3	2010	1500	(48)
Lack of sexual satisfaction	higher education, working mothers, marriage age	_	2009	618	(50)
Lack of sexual satisfaction	higher education, working mothers	_	2013	200	(52)
labor injuries		40	2005	13117	(56)
	Fetal distress, lack of labor progression, maintaining the health of both the mother and fetus, mother request, multiple pregnancy, mother disease, private hospitals, high pregnancy age, low pregnancy age	51.6	2013	103348	(59)
	Fetal distress, lack of labor progression, previous Cesarean , abnormal appearance, mother request	32.6	2013	950	(60)
	Lack of labor progress, fear of pain, doctor recommendation, request mother	58.6	2011	396	(61)
Apgar score below 7		13	2010	294	(62)
	Abnormal appearance, repeated Cesarean , fear of pain, multiple pregnancy, high pregnancy age, higher education, history of infertility,CPD, history of abortion or still birth, history of maternal hypertentation	59	2012	200	(63)
	Failure of labor progression, fetal distress, abnormal presentation, previous Cesarean , tubal ligation, multiple pregnancy, history of ,infertility, CPD , decrease fetal movement	63.3	2012	600	(64)
	Private hospitals , Class status	26.1	2002	5874	(65)
Average maternal admission		86	2007	411	(66)
RDS, abnormal bleeding, labor injuries	Lack of progress of labor, fetal distress, abnormal presentation	19.1	1999	5440	(67)
Fever, muscular pain, headache, infections, digestive problems		48	2010	300	(68)
TTN, RDS	High pregnancy age, low pregnancy age, working mothers	50	2005	600	(69)
TTN, RDS, NICU admission, Apgar score below 7	Abnormal appearance, previous Cesarean , mother request, high pregnancy age, low pregnancy age, higher education	_	2013	250	(70)
Abnormal bleeding, fever, digestive problems, average maternal admission		73	2004	593	(71)
Abnormal bleeding, fever, infections, digestive problems, average maternal admission, Apgar score below 7	Fetal distress, lack of labor progression	68	2005	253	(72)
SUI	Lack of labor progress, maternal request, mother disease	49.1	2006	702	(73)
Average maternal admission		42	2014	536	(74)
RDS		20	2005	170	(75)
	Lack of labor progress, fetal distress, previous Cesarean , mother request, multiple pregnancy, private hospitals , CPD , Class status , Recommended acquaintances	45	2012	24241	(76)
	Lack of labor progress, mother request, previous Cesarean , tubal ligation, high pregnancy age, low pregnancy age, higher education , CPD	44	2004	13599	(77)
	Mother request, high pregnancy age, low pregnancy age, higher education, working mothers ,the husband request	41.4	2013	384	(78)
Average maternal admission	Higher education, working mothers	49	2013	1172	(79)
	Fetal distress, lack of labor progression	26	2011	1537	(80)
Lack of sexual satisfaction	previous Cesarean , fear of pain, doctor recommendation, tubal ligation, private hospitals, higher education, working mothers , Lack of knowledge	_	2006	60	(81)
Abnormal bleeding, digestive problems, SUI, muscular pain, headache	High pregnancy age, low pregnancy age, higher education, working mothers	61	2012	308	(82)
SUI		71	2008	1400	(83)
SUI		16	2001	400	(84)
Abnormal bleeding, digestive problems, labor injuries, SUI, RDS, NICU admission	Higher education, working mothers	46	2012	246	(85)
Lack of sexual satisfaction	Higher education, working mothers	_	2012	366	(86)
Lack of sexual satisfaction	Higher education, working mothers	_	2011	180	(87)
Lack of sexual satisfaction	High pregnancy age, higher education, working mothers	_	2010	280	(88)
SUI		16	2001	400	(84)
Abnormal bleeding, gastrointestinal problems, SUI, RDS, labor injuries, hospitalized in the NICU	Higher education of mother, working mothers	-	2012	246	(85)
sexual dissatisfaction	Higher education of mother, working mothers	-	2012	266	(86)
sexual dissatisfaction	Higher education of mother, working mothers		2011	180	(87)
sexual dissatisfaction	Maternal age, education, working mothers		2010	280	(88)
	Location, lack of awareness, marriage age, class status, history of infertility, miscarriage and stillbirth, CPD, advised acquaintances	47	2012	600	(89)
	Fear of pain, doctors recommendation, repeated Cesarean section, maternal request, Parity, Location, abortions and stillbirths, the wife request	57	2013	450	(90)
	Maternal request, abnormal presentation, location, Age at marriage, abortion and stillbirth, CPD	37	2000	500	(91)
	Fear of pain, lack of awareness, class status, spouse's education, marriage age	19	2015	196	(92)
	Fear of pain, doctors recommendation, mother request, lack of awareness, class status, place of residence, the husband request, relatives advised	-	2016	739	(93)

## Discussion

The results of the present study indicated that the rate of cesarean prevalence in Iran was estimated at 48%. 

Higher education, mothers’ employment, high pregnancy age was the most important socio-demographic factors for the prevalence of cesarean delivery. Previous cesarean and fetal distress were also the most significant obstetrical-medical reasons for cesarean delivery. Doctor recommendation and fear of labor pain were also identified as the most important non-obstetrical and non-medical reasons for cesarean delivery. Among individual and social factors affecting cesarean, education, employment and maternal age had the greatest impact. Azami-aghdash *et al* reported the prevalence and causes of cesarean section in Iran about 48%. Their study included 34 articles in a meta-analysis model, while 41 articles are included in the meta-analysis model of this study, The search period was also from 1999 to 2016 vs. Azami-Aghdash’s one which was from 2000 to 2012 (24). Since the prevalence of cesarean was estimated based on the time course and province, the analysis of the subgroup for the time period showed that in the period of 2005-2009, the most prevalent cesarean section (53%) occurred in the country. The lowest prevalence of cesarean delivery was also found to be 19% between 2015 and 2017, which is largely due to the small number of studies carried out during that period. In the study of subgroup analysis based on the province of the study, Isfahan province with (76%) had the highest prevalence of cesarean section and Hormozgan province with (24%) had the lowest prevalence of Cesarean section. The interpretation for this difference in result depends on a number of factors, including socio-cultural differences and economic aspects in different parts of the country. Also, the difference in access to health care services, as well as the quantity and quality of prenatal care in different regions of the country, is effective in this difference.

In this study, the prevalence of C-section in Iran was 48%, which is similar to its corresponding result from the previous meta-analysis study. High education with 16 and 15-repeat and mother’s employment are considered as the most important individual and social factors in the prevalence of cesarean section in the reviewed articles. The high age of the mother (over 35) is also another factor affecting the prevalence of C-section. In the previous meta-analysis study, higher education and high maternal age were the most important personal and social factors affecting the prevalence of cesarean section.

The result of this study showed that repeat cesarean section with an average of 42.25% and fetal distress with an average of 22.11% are the main reasons for C-section. Also, among the medical reasons, the decrease in fetal movements with an average of 2.1% and multiple pregnancies with an average of 3.2% are the least effective factors on cesarean section. In the previous meta-analytic study, whereas the main reasons for C-section were repeated cesarean section with an average of 36.29%.

The result showed that, physician's recommendation with an average of 38.8% and fear of vaginal delivery pain with an average of 34.62% are the most important non-medical factors affecting the prevalence of C-section. In the previous meta-analytic study, the most common non-medical reasons for C-section were fear of vaginal delivery pain with an average of 39.33% and a physician's recommendation with an average of 28.45%.

Studies have shown that cesarean rate has been growing dramatically in Iran during the recent years. This increase is also seen in different parts of the world (28). According to a study conducted in the UK, one of every five pregnant women has a cesarean delivery; while the maximum cesarean rate in the country was only 4 percent 30 yr ago (29). The rate of cesarean in Iran is reported from 26- 66.5% by various studies, while it’s reported as even 87% by some private centers (8, 9). Such a wide range and outstanding differences could be due to several factors, including cultural differences, social and economic aspects in different areas of the country.

Different studies have reported different statistics regarding the prevalence of cesarean in different regions of Iran. The differences in the amount of access to health services as well as quantity and quality of prenatal care can also have a major influence on the prevalence of cesarean in different parts of the country. 

The results of the articles reviewed in this study showed that cesarean rate among the women with higher education were higher than that of the women with lower education. Various studies have shown that the most effective option in their decision making regarding the mode of the delivery, is Obstetricians and Gynecologists (30, 31). It does not seem, however, that this is a direct result of an increased awareness; rather, it is due to the fact that with increasing levels of education, people are more likely to have better job opportunities and income, which will lead to a socio-economic growth, and then cesarean increase rate is socially regarded as a sign of higher social status and mother’s convenience. The results of the study showed that cesarean rates were higher in private hospitals than in public hospitals in such a way that 57.7% of the cesareans were carried out in private hospitals and 33.6% in public hospitals. Perhaps the reason for this dissimilarity is the difference between cesarean delivery financial tariffs and those of normal vaginal delivery, especially in the private centers, which will reflexively lead the doctors towards cesarean delivery. It can be said that the economic status of those who admit to private hospitals has resulted in a dramatic increase in the cesarean rates in these centers (31). 

According to the results obtained in this study, doctor recommendation is an important reason for choosing cesarean delivery. In 70% of the cases, the doctor plays a major role in determining the delivery method (32). The role of doctors in increasing the cesarean rates in the past decades has been to such an extent that some researchers have regarded doctors’ medical judgment and their environmental conditions -not the patients’ medical condition- as the main reason for cesarean decisions (33, 34). Studies have shown that the most important reason for doctors’ unnecessary offers to perform caesarean were doctors’ lack of sufficient expertise in dealing with some demanding vaginal deliveries and doctors’ tendency to spend less time and earn more money performing delivery operations (35).

The results of the studies included in the present study indicated that the previous cesarean was the most significant obstetrical-medical reason for the incidence of cesarean delivery, which is consistent with the findings of most studies done in this area (13, 36-39). Most women, who have their first delivery by cesarean, may choose the same method for their subsequent delivery; and this would lead to an increase in cases of caesarean sections in the future (40). Therefore, through proper planning and awareness-raising during pregnancy, especially in primiparous women, a long step in maternal and neonatal health can be taken because of the fact that planning on primiparous women will affect the labor process in the future. It can also prevent the high cesarean rate due to previous cesareans in the future. 

Review articles in this study showed that the cesarean rate among women with a higher education higher than women with lower education. Results of other studies, such as Ahmadnia and Murray showed an increase in maternal education level, C-section rates increased (40, 41). It seems that it is for this reason that increasing the level of education, job and income opportunities for people to be better, This increases the level of economic and social status And because C-section in terms of the social bookmarking higher status and be considered a sign of respect to comfort the mother, it contributes to an increase in caesarean sections. In this regard, it seems that high levels of C-sections tend to be employed in women can be due to the higher education and better economic situation of them (42). The results showed that the rate of C-section in private hospitals (57.7%) higher than public hospitals (33.6%), perhaps because of these financial differences between cesarean section and vaginal delivery tariffs, especially in the private centers that this practice unwanted leads to do by doctors, it can be said that differences in Economic status that people referred to private hospitals in these centers had been an increase in gross cesarean rate (43).The results showed a lack of awareness about delivery methods affect the choice of delivery method is such that 64.8% of women had a very low information about delivery, Studies have shown that raising awareness to change attitudes and ultimately change their health behavior, Therefore raising awareness of pregnant women about the complications and benefits of cesarean and vaginal delivery is effective first step to reduce caesarean sections without medical indication (44, 45). This study showed that individual and social variables, number of pregnancy have minimal impact on the incidence of cesarean section. Studies have shown that the average number of pregnancies for women with cesarean delivery was lower than women with vaginal delivery and cesarean section rate in nulliparous women is more than the other(46, 40).

Investigating the labor maternal complications in this study showed that post-delivery problems in women who had undergone cesarean were more than those of the women who had a labor through normal vaginal delivery. Such complications as muscular pain, headache and fever were observed more in the cesarean mothers, part of which can be attributed to anesthesia methods used in caesarean section (47, 48).

Today, researchers believe that one of the important reasons for women’s tendency towards cesarean is the negative impact they assume for the normal vaginal delivery to have on their sexual function after the delivery (49,50). This study revealed that frequency of lack of sexual satisfaction after delivery was higher in cesarean mothers than in women who had had normal vaginal delivery. Studies have shown that not only a safe vaginal delivery with a minimum of perineum trauma is not associated with sexual dysfunction, but also can lead to better sexual relations with the partner (51). Considering to the cultural conditions of the society and the importance of sexual satisfaction in marriage with an emphasis on the delivery method, the importance of a safe vaginal delivery with minimal trauma should always be taken into account. Moreover, training, proper consultancy, providing the couples with the appropriate information before, during and after the pregnancy is crucial.

Of the reasons for cesarean delivery, one is the belief that cesarean delivery would protect the health of the fetus. Neonatal complications in this study showed that respiratory disorders (TTN & RDS) were more prevalent among the newborns by Cesarean than among the newborns by vaginal delivery. Respiratory problems are of the main reasons for the newborns’ morbidity and mortality (46).Different studies have shown that respiratory complications have significantly been higher in newborns with cesarean. The risk is doubled for the caesarean deliveries performed before week 39 and especially for the mothers who had not experienced labor pain (52-54). Hence, experts recommend that for the sake of the newborns’ health, no cesareans be carried out without obstetrical indications and that cesareans are performed only in case of emergency and after the onset of delivery pain (53). 

The results of the articles reviewed in this study revealed that cesarean has an important role in the prevention of labor injuries and trauma. Such traumatic complications as broken bones, cephalhematoma and obstetric asphyxia are more reported in many studies for vaginal delivery than caesarean (55-57), the reason for which can be lack of sufficient expertise for performing the normal vaginal delivery (17). Accordingly, to prevent any harm to the newborns and to amend the society's attitude towards normal vaginal delivery, fostering the awareness and the skills of the delivery operating personnel as well as improving the facilities and equipment is of absolute necessity. 

The strength of this study is that updates the previous article, also we have included a new analytical part for the effect of the time course and province

## Conclusion

Regarding its adverse effects on maternal and neonatal health and the health system as well, the intense and increasing of the prevalence of cesarean in Iran has become a pervasive problem. Hence, to reduce the cesarean rate and to direct the mothers towards normal vaginal delivery, providing practical solutions to the health system planners and authorities is of great significance. Given that the previous cesarean, doctor recommendation, fear of labor pain, and protecting the health of the fetus are the main reasons for cesarean delivery, such measures as proper training of the techniques for pain control and pain reduction, improving the quality of normal vaginal delivery services, culture-building practices for normal vaginal delivery, making women aware of the complications of cesarean delivery, and establishing a mechanism to halt the enforcement of doctors’ personal opinion can be beneficial. 

## Conflict of interest

There are no benefits in any form have been or will be received from a commercial party related directly or indirectly to the subject of this article.
